# The spermatozoa caught in the net: the biological networks to study the male gametes post-ejaculatory life

**DOI:** 10.1186/1752-0509-4-87

**Published:** 2010-06-18

**Authors:** Nicola Bernabò, Mauro Mattioli, Barbara Barboni

**Affiliations:** 1 Department of Comparative Biomedical Sciences, University of Teramo, 64100 Teramo, Italy

## Abstract

**Background:**

Mammalian spermatozoa, immediately after the ejaculation are unable to fertilize the oocyte. To reach their fertilizing ability the male gametes must complete a process of functional maturation, the capacitation, within the female genital tract. Only once the capacitation is completed the spermatozoa can respond to the oocyte interaction with the exocytosis of acrosome content, acrosome reaction (AR). These post-ejaculatory events are under the attention of Researchers from more than fifty years but their basic knowledge is still unsatisfactory. This failure could be due not to the insufficiency of available data, but to the inability to manage them in a descriptive model. Thus, to overlap this problem, the capacitation and the AR were represented using the biological networks formalism. In addition the effect of elimination from both the networks of the most linked (the hubs) or of random selected nodes was verified and the network representing the common element of capacitation and AR (C∩A) was realized.

**Results:**

The statistical analysis of resulting graphs showed that capacitation, AR and C∩A networks follow the scale free topology and are characterized by low clustering. In all cases it was possible to identify the key molecules (Ca^2+^, ATP, P-Tyr, PKA, PLD1 in capacitation, Ca^2+^, ATP in AR and C∩A) and to describe their role in signalling transduction. The effect of hubs elimination caused the collapse of networks structure, while the elimination of random selected nodes did not affected it.

**Conclusions:**

It was demonstrated that the post-ejaculatory life of male gametes is a series of events characterised by a high signalling efficiency and robustness against random failure. This strengthens the evidence that the adoption of biological networks modellization of capacitation and AR could increase the understanding of spermatozoa physiology, potentially opening new perspective in drug discovery, diagnosis and therapy of male infertility.

## Background

The latest years are characterized by the amazing diffusion of biotechnologies of reproduction. In a relatively short period, many techniques have been developed and routinely introduced in Assisted Reproductive Technologies [1]. Unfortunately, this applicative knowledge is not paralleled by a correspondent increase in the understanding of the basics of reproductive biology. In fact, during the organism's life, the gametes undergo important processes of proliferation, differentiation and morpho-functional maturation whose biochemical and physiological determinants are still largely unknown. This is the case of the mammalian spermatozoa that, once ejaculated, are not immediately able to fertilize. Only after the interaction with the female genital tract for a relatively long period, from hours to days, depending on the species, these cells can recognize and bind the proteins of the oocyte glycoprotyeic coat, the zona pellucida (ZP). The physico-chemical modifications of spermatozoa (collectively known as "capacitation") involve the biochemical and biophysical asset of the whole sperm cell. Overall the variations of the extracellular milieu, from the epididimis and seminal plasma to the female genital tract secretions, induces the membrane receptors activation that lead to the intracellular signal transduction. These signalling pathways drive the cellular response: the intracellular calcium concentration rises [2,3], the protein phosphorylation pattern changes [4,5], the actin cytoskeleton reorganizes [6,7], the plasma membrane (PM) and the outer acrosome membrane (OAM) become more instable and gradually acquire the ability to fuse each other [8,9], the spermatozoa motility is hyperactivated [10]. Once completed, the capacitation makes the spermatozoa able to recognize, to bind and to interact with the zona pellucida of oocytes. In this case also, the male gamete undergoes to important morpho-functional modifications culminating in the vesciculation of PM and OAM and in the release of acrosome content (acrosome reaction, AR) [11]. After the completion of these preliminary phases the spermatozoa can reach the perivitelline space, fuse with oocytes membrane and the fertilization starts.

The amount of data available on mammal and, in particular, on human capacitation and AR is really impressive. At the present (9/10/2009) the peer-reviewed accessible papers on PubMed database typing "capacitation" are 3434 (495 reviews) and "human capacitation" are 1655 (355 reviews); typing "acrsome reaction" are 3137 (362 reviews) and "human acrosome reaction" are 1511 (268 reviews). Despite this large amount of literature, many aspects of these events are still unsatisfactory known. In particular, this deficiency is evident in diagnosis, therapy and prognosis of several human (and animal) pathological conditions. It is, in fact, universally accepted that, at the present, a marker that univocally *a priori *predicts the spermatozoa ability to fertilize does not exist. At the same time, in a relevant percentage of cases, it is impossible to perform a diagnosis (and as a consequence a therapy and a prognosis) after seminal and clinical investigations, as it happens in the unexplained infertility [12] of male origin. It is possible to conceive that the lack of knowledge could not be due to the insufficient amount of data but to the inability to manage them in an appropriate explicative model. Thus, aim of the present research, was to build a descriptive model focusing not only on the molecules involved in spermatozoa physiological maturation, but examining the complex of their interactions adopting the graph theory formalism. In fact, a system of elements that interact or regulate each other (as it happens during capacitation and AR) can be represented by a mathematical object called "graph" [13]. The graphs are constituted by a variable number of nodes (the molecules) linked by edges (interactions) originating a network. The edges can be undirected or directed, as in the case of metabolic and signal transduction pathways, specifying a source (starting point) and a target (endpoint), representing the mass or information flow [14]. This approach was adopted to increase the understanding of pivotal biological events, such as the capacitation and the AR, representing them as complex entities, whose behaviour is determined by the molecular components and by their interactions. In this context it is important to note that the system theory stated that in complex systems, such as the cell, the whole system is more than the sum of its single components.

## Results

### Capacitation and AR networks

**Figure 1:**
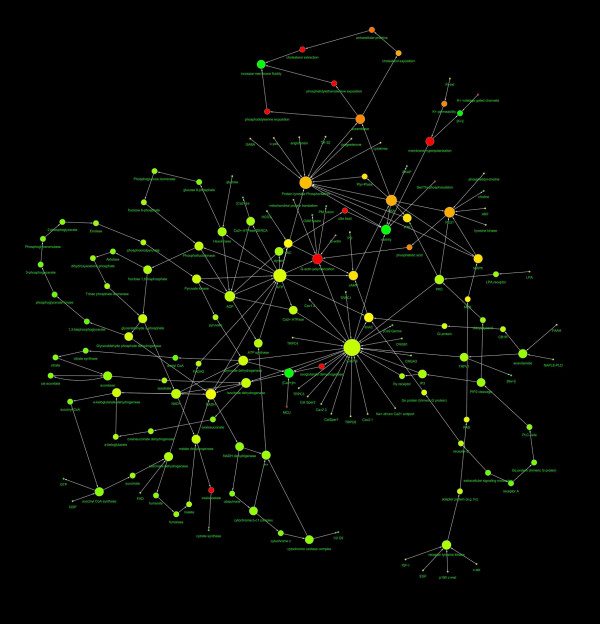
**Diagram showing the structure of the capacitation network**. The nodes diameter is proportional to the number of links, the color varies depending on the network centrality. The direction of arrows represents the direction of the interaction (from the source to the target). The spatial network arrangement was obtained by using the Cytoscape Spring-embedded Layout (see the text for explanation).

**Figure 2:**
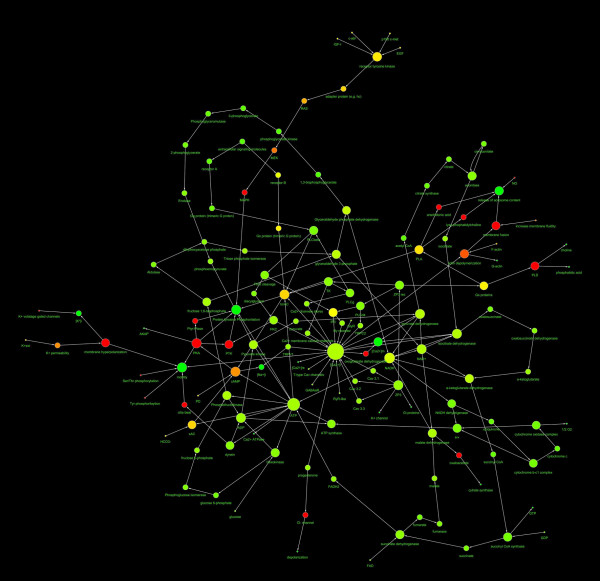
**Diagram showing the structure of the AR network**. The nodes diameter is proportional to the number of links, the color varies depending on the network centrality. The direction of arrows represents the direction of the interaction (from the source to the target). The spatial network arrangement was obtained by using the Cytoscape Spring-embedded Layout (see the text for explanation).

The network representing the capacitation, and the AR of human spermatozoa were represented in Fig. 1 and Fig. 2 and the main topological parameters of all the networks were showed in Table 1. In both cases the distribution of node linkages followed a power law, represented by the generic equation:

**Table 1:** Main topological parameters of capacitation and AR networks

	capacitation	AR
N°nodes	146	141
N°edges	197	191
Clustering coefficient	0.029	0.026
Diameter	20	20
Averaged n°neighbours	2.667	2.695
Char. path length	6.606	6.736

The number of nodes represent the total number of molecules involved, the number of edges represents the total number of interaction found, the clustering coefficient is calculated as *C*I = 2*n*I/*k*(*k*-1), where *n*I is the number of links connecting the *k*I neighbours of node I to each other, the network diameter is the largest distance between two nodes, the Averaged n°neighbours represent the mean number of connection of each node, the Char. path length gives the expected distance between two connected nodes.

The r, R^2 ^and b coefficients of each network were tabulated in Table 2.

**Table 2:** Result of power law fitting of IN and OUT capacitation and AR networks

	capacitation		AR	
	in	out	in	out
r	0.992	0.997	0.992	0.989
R^2^	0.897	0.837	0.906	0.823
b	-1.547	-2.046	-1.657	-2.303

The clustering coefficient distribution does not follow a power law, thus the results of power law fitting of clustering coefficient distribution were: capacitation: r = 0.152, R^2 ^= 0.194; AR: r = 0.023, R^2 ^= 0.132.

The most connected nodes in both the networks have been identified as depicted in Table 3.

**Table 3:** Most connected nodes (the hubs) of capacitation and AR networks

Network	Node	Number of links
capacitation	[Ca^2+^]_i_	25
capacitation	ATP	14
capacitation	Tyr phosphorylation	13
capacitation	PKA	9
capacitation	ADP	8
capacitation	PLD1	8
AR	[Ca^2+^]_i_	23
AR	ATP	13

The percentage of one-link nodes ranged from 22% (AR) to 30% (capacitation), while about the 40-45% of nodes had two links.

### Networks modification

**Figure 3:**
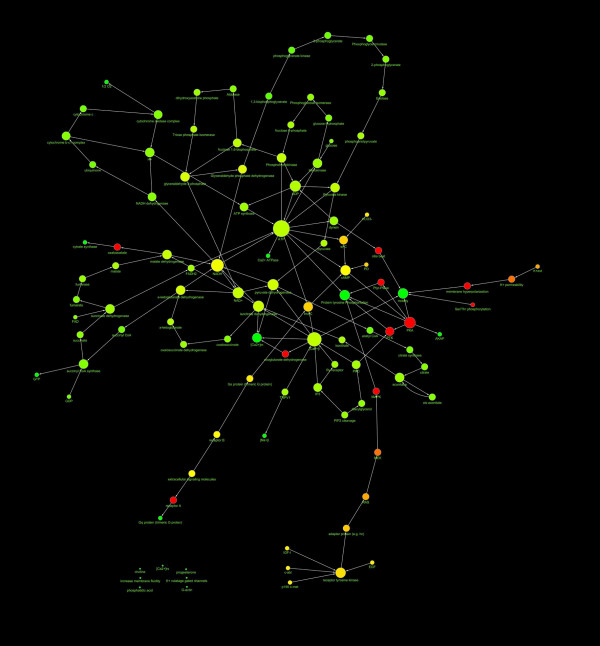
**Diagram showing the structure of the C∩A network**. The nodes diameter is proportional to the number of links, the color varies depending on the network centrality. The direction of arrows represents the direction of the interaction (from the source to the target). The spatial network arrangement was obtained by using the Cytoscape Spring-embedded Layout (see the text for explanation).

The network representing the intersection of capacitation and AR networks (C∩A) was realised (see Fig. 3). Their main topological parameters are listed in Table 4:

**Table 4:** Main topological parameters and the most connected nodes of C∩A network

	C∩A
N°nodes	109
N°edges	143
Clustering coefficient	0.036
Diameter	20
Averaged n°neighbours	2.606
Char. path length	6.957
IN degree distribution	b = -1.829
	r = 0.997 R^2 ^= 0.948
OUT degree distribution	b = -2.240 r = 0.992 R^2 ^= 0.894
Hub (n°edges)	ATP (13); Ca^2+ ^(12)

The number of nodes represent the total number of molecules involved, the number of edges represents the total number of interaction found, the clustering coefficient is calculated as *C*I = 2*n*I/*k*(*k*-1), where *n*I is the number of links connecting the *k*I neighbours of node I to each other, the network diameter is the largest distance between two nodes, the Averaged n°neighbours represent the mean number of connection of each node, the Char. path length gives the expected distance between two connected nodes.

The power law fitting values for clustering coefficient distribution were: r = 0.027 and R^2 ^= 0.005.

**Figure 4:**
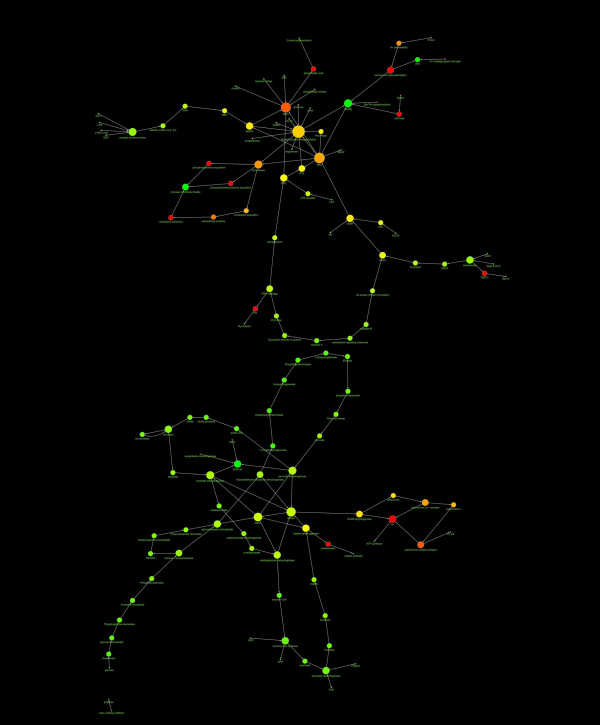
**Diagram showing the effect of the elimination from capacitation network of the most linked nodes**. The elimination from capacitation network of the most linked nodes ([Ca^2+^]_i _and ATP-ADP) caused the collapse of network structure.

**Figure 5:**
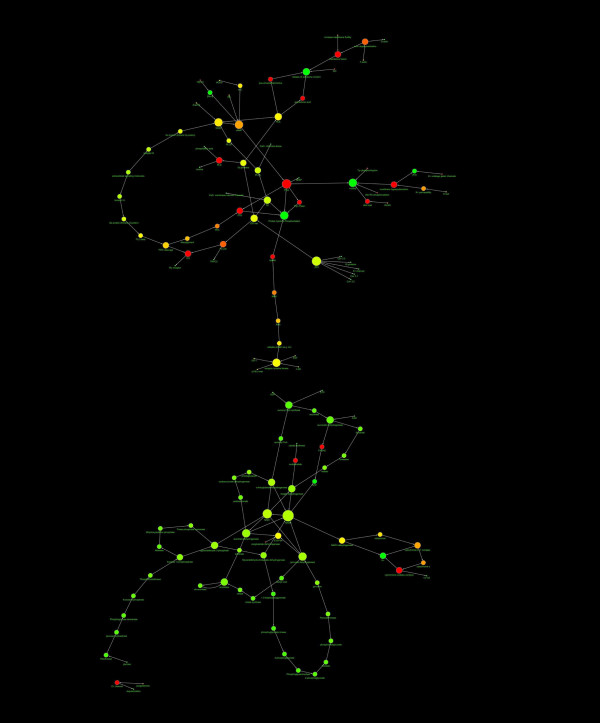
**Diagram showing the effect of the elimination from AR network of the most linked nodes**. The elimination from AR network of the most linked nodes ([Ca^2+^]_i _and ATP-ADP) caused the collapse of network structure.

The [Ca^2+^]_i _and the ATP-ADP system or 2 or 4 random nodes were eliminated from the capacitation and AR databases to examine the effects of targeted or random deletion of nodes from the networks. As a result in the first case, both in capacitation and AR networks, the ablation of hubs determines the destruction of networks: the networks were cut in two independent structures and their architecture collapsed (see Fig. 4 and Fig. 5).

In the cases of elimination of randomly individuated nodes the networks architecture and topology does not changed significantly (data not shown).

## Discussion

Aim of this work was to represent and to study the biochemical events occurring during sperm capacitation and AR using the formalism of networks theory to improve the knowledge about these important steps of the fertilization process. The main problem related to this approach was the unavailability of specific databases of events. Thus we ex novo built them, starting from the data available on scientific literature (PubMed). In any case it was adopted a cautious approach: only pluri-referenced recent data, in particular if referred to human, were adopted. Moreover, it was impossible to calculate if the percentage of molecules included in the database was representative of all biochemical asset of spermatozoa: the total number of different molecules present in sperm cell is, at the present, unknown.

Anyway, the study of topological proprieties of networks allowed the characterization of some important aspects of sperm post-ejaculatory life. In particular, the networks were classified on the basis of their topological proprieties [15]. The most elementary characteristic is the node degree (or connectivity), *k*, which indicate how many links the node has to other nodes. As a consequence it is possible to define the node degree distribution, *P*(*k*), which represents the probability that a selected node has exactly *k *links. In addition the network tendency to develop clusters of nodes can be quantified by using the clustering coefficient *C*I = 2*n*I/*k*(*k*-1), where *n*I is the number of links connecting the *k*I neighbours of node I to each other. More the clustering coefficient is high, more the presence of clusters increases. Overall three different classes of networks were defined: random networks, scale free networks and hierarchical networks [15]. The random networks were described by the Erdös-Rényi (ER) model that imply that the node degrees follows a Poisson distribution, thus the most of nodes have approximately the same number of links (that defines the network's scale). Moreover, the clustering coefficient is independent of the nodes degree. The scale-free networks (Barabási-Albert, BA, model) are characterized by a power-law degree distribution of the number of links per node. Thus, a relatively small number of nodes is highly connected (the hubs) and most of the nodes are scarcely linked. As a consequence it does not exist a "typical" node (scale free topology). In addition the clustering coefficient is independent of the number of links per node. In the hierarchical networks the scale-free topology and the local clustering coexist. In this case the clustering coefficient is higher in the most linked nodes and, consequently, its distribution follows a power law.

The analysis of the main topological parameters carried out on the capacitation and AR networks showed that these networks were referable to the scale-free networks, as demonstrated by the power law that links the number of edges to the node frequency and the dispersion of clustering coefficient in agreement with the BA model. Consequently, it was possible either in the case of capacitation and AR networks to individuate the most linked nodes (the hubs).

It is of high interest to note that the hubs in case of capacitation are, the [Ca^2+^]_i_, the ATP-ADP system, the protein kynase A (PKA), the Tyr phosphorylation and phospholipase D1 (PLD1).

It is known that during capacitation the [Ca^2+^]_i _increases, in response to control factors and that the capacitation does not take place in the Ca^2+ ^absence [2,11]. At the present four major Ca^2+ ^clearance mechanisms are described in mammalian spermatozoa, two acting on the plasma membrane and two on intracellular organelles. The plasma membrane Ca^2+^-ATPase exports a cytoplasmic Ca^2+ ^ion and imports one or two extracellular protons at the expense of ATP. When [Ca^2+^]_i _is elevated, the plasma membrane Na^+^-Ca^2+ ^exchanger operates in forward mode exporting an intracellular Ca^2+ ^ion and importing approximately three Na^+ ^ions at the expense of the Na^+ ^gradient [16,17]. The best characterised organellar clearance mechanism is the sarcoplasmic-endoplasmic reticulum Ca^2+^-ATPase pumps and the mitochondrial Ca^2+ ^uniporter [18]. During the sperm capacitation the Ca^2+ ^behaves like a second messenger converting extracelluar stimuli to chemical response in a myriad of molecular system, such as, protein kynase C (PKC), protein kynase C (PKA), actin, and many others.

The presence of the ATP-ADP system in both the networks could be explained considering that ATP is the main energetic source of spermatozoa. In fact in this cell, the metabolic energy production is fuelled by the glycolysis exclusively, by mitochondrial oxidative phosphorylation exclusively, or by a combination of both the pathways [19].

The PKA is involved in several biochemical events, in different pathways. The HCO_3_^− ^and Ca^2+ ^are transported by a Na^+^/HCO_3_^− ^cotransporter (NBC) and a sperm-specific Ca^2+ ^channel (CatSper) and promote, via soluble adenylyl cyclase, the PKA activity. In addition it is involved in late events that lead the spermatozoa to acquire hyperactivated motility. At the molecular level, these changes are correlated with an increase in tyrosine phosphorylation dependent on the presence of cholesterol acceptors in the capacitation medium exerted by PKA [20,21].

Protein tyrosine phosphorylation pattern of spermatozoa changes during the capacitation in several species such as human, mice, cattle, pigs, hamsters and cats [4,22,23]. This process appears to be a necessary prerequisite for a spermatozoon to fertilize an egg and has been demonstrated to increase both in flagellum and in the sperm head, but at present the molecular target of protein tyrosine phosphorylation are largely unknown [23].

The PLD1 plays a key role in the polymerization of actin that is one of the most important events occurring during capacitation. More in particular MAP-kinase, tyrosine kinase, and ADP-ribosylation factor are involved in PLD activation, leading to phosphatidyl-choline hydrolysis to produce phosphatidic acid, which mediates polymerization of G-actin to F-actin [24-26].

The hubs found in the AR process are the [Ca^2+^]_i, _and the ATP. It is known that during the AR one of most relevant events is the very fast surge of [Ca^2+^]_i_, following the spermatozoa interaction with oocyte. This ionic event is due to the opening of voltage gated calcium channels (VGCC) as a consequence of the membrane depolarization wave. The dependency of AR from calcium metabolism is strengthened by the concept that spermatozoa are calcium-dependent cell, such as neurons [2,27].

The hubs found in capacitation and AR networks reflect the differences in signalling strategy during the corresponding biological events. In both cases the ADP-ATP system is the energy supplier. The capacitation, that is a time consuming multi-step process, requires the activation of several biochemical signal transducers (ions, protein conformation variation, lipid remodelling) to orchestrate the maturation events involving all the subcellular components (membrane, flagellum, mitochondria, cytosckeleton). The AR, on the contrary, is characterized by a peak of [Ca^2+^]_i _that causes a rapid and ubiquitous cascade of reactions that lead the spermatozoa to the rapid and irreversible loss of acrosome content. In addition the idea that the spermatozoa are calcium-dependent cells is strengthened by this finding.

It is interesting to note that the most hubs of A∩C were ATP and Ca^2+ ^but, unlike in the case of capacitation and AR, the most connected is ATP and Ca^2+ ^had 12 links only. This finding is justified by the concept that the A∩C represent the backbone of sperm metabolism, thus the energetic supply is the most important function. As confirmation it is noteworthy that ATP in all the networks (capacitation, AR and A∩C) had virtually the same connectivity (14, 13 and 13 nodes respectively) and interacted with the same molecules.

Moreover it appeared logical that the Ca^2+^, which is a signal transduction molecule, was less connected in A∩C network than in the others networks, whose physiological meaning is the acquisition and completion of signalling cascades. Unlike the ATP in the case of Ca^2+ ^the number of links was different (capacitation: 25, AR: 23) and only the 50% are common to capacitation and AR (A∩C: 12).

In addition, the network's statistical analysis point out that the value of clustering coefficient was very low in cap and AR networks, while was higher in A∩C network. The cap and AR are chains of signalling events, as a consequence the molecular message must be carried, directionally, from the beginning to the end of the chain (this is also the reason why the averaged number of neighbours in about 2: one is the input and the other is the output of message). In this optic the presence of loop or clusters could interfere and slow the propagation of messages. On the contrary, A∩C network represents the common motifs present in cap and AR, that is the metabolic scaffold of spermatozoa. Thus it is not surprising that the clustering coefficient is higher because of the presence of metabolic loops.

The characteristic path length, also, (always ranging from 6.6 to 6.8) may have two important consequences on spermatozoa physiology. Firstly if any molecule can interact with any other in a small number of passages, the loss of information due to the signal decrease is minimized and, consequently, the signal efficiency is maximized. In addition any local perturbation in signalling system could reach the whole network in a short time, thus increasing the cellular system responsiveness to the intracellular and extracellular stimuli. From these data emerges the strong link between the sperm signalling machinery and their function, emphasized by the study of metabolic architecture concretely depicted by the networks model.

When two random selected nodes were eliminated the variations of networks stability and topology were minimal. On the contrary, when the most connected nodes ([Ca^2+^]_i _and the ATP-ADP system) where eliminated, the networks structure collapsed. This finding is in perfect agreement with the theoretical consideration on robustness of scale free networks [28,29] and with the experimental data, unequivocally demonstrating that in the absence of Ca^2+ ^or of the ATP-ADP system the capacitation and the AR do not take place [30]. As a consequence it emerges that the model structure seems to be highly predictive of real situations. From an evolutionary point of view it is possible to speculate that in spermatozoa, which are indispensable for species survival, the scale free architecture is evolved because of its high degree of robustness against a random damage. The random removal of nodes will take out mainly the less linked ones because they are the most frequent. In this case the cellular function will be unaffected by the damage. Vice versa, the hubs are the less frequent nodes, thus the probability that their failure will happen, is low.

It was possible to isolate the nodes with only one link. The classical scale free model indicate that these nodes are the most periferical and, as a consequence, less important ones. For instance in the WWW and internet the sites with only one link are the most marginal in the network and, as a consequence are destined to disappear [31]. In the present case, the nodes with one link are, in most cases, the input terminal of network. In fact they linked the intracellular signalling pathway. In the real situation, putting the spermatozoa in their environment, these nodes could be able to link and to interact with the external molecules. In other words the one-link nodes are the input terminal of the system and are connected with the output terminal of contiguous systems (female environment, seminal fluid, artificial media in laboratory technologies, ...).

It is of interest, also, to note that almost half of the nodes (40-45%) have two links. This is in agreement with the concept that the networks had a signalling transduction-dedicated structure: each node receives an input signal and transfers an output response. In addition the terminal events (such as protein phosphorylation or membrane fusion) are highly linked. Reasonably this is due to the redundancy of biochemical signalling, as a safety strategy to overlap partial failure of the system.

On whole it is evident that the activating signals are markedly most expressed than the inhibiting ones (~95% vs. ~5%). This evidence could have two different explanations:

- it is possible that the interest to recreate in vitro the sperm capacitation in the contest of Assisted Reproductive Technologies lead the researchers to study and describe the capacitation-promoting events. Thus the activating signals are not the most expressed but the most studied and, as a consequence, the most of information available on literature are concerning these events;

- the spermatozoa are functionally disposable cells. From a teleological point of view their fate is the completion of capacitation, acrosome reaction, and, after all, the fertilization. Thus, it is possible that most of the biochemical pathways are objective-oriented. In fact these cell must not maintain the cellular homeostasis for long time but must be able to respond to the activating stimulus.

This work, for the first time, allows the representation and the study of the important steps of spermatozoa functional maturation, which happens within the female genital tract, using the biological networks formalism. The data used to this aim are prevalently referred to human. Since the capacitation is a species-specific process, it is possible to hypothesize that the corrisponding models in different species could be more or less different too. This could open the perspective to use the computational modellization of post-ejaculatory maturation of spermatozoa from different species to study the biochemical and physiological analogies and differences. This, potentially, could lead the increase in comparative and evolutionary knowledge of this pivotal event.

For many reasons these cells are an ideal candidate for this kind of analysis:

- first of all they are virtually transcriptionally silent, as a consequence their protein composition is stable. Indeed the most important problem in cell modelization is the continuous modification in cellular protein content and in molecular interactions due to the dynamical regulation of genes expression and protein transcription.

- differently from the most of other cellular types, it is possible to empirically evaluate the functional status of the system. In fact it is possible, using for instance an animal model, to verify if the spermatozoa completed their maturation process, testing the ability of spermatozoa to complete the capacitation and, subsequently, to undergo AR by in *in vitro *fertilization assay or by *in vivo *fertilization trials: only the spermatozoa that successfully fertilize an oocyte can be considered fully competent.

- finally the spermatozoa are the only cellular type, produced in an organism, that exert their function in another one. As a consequence they are capable of independent life (unlike the other cells) and it is possible to manage them outside the organism without loss of the cell function.

Moreover it is possible to hypothesize that the future and continuous upgrade of biochemical data databases could improve the effectiveness of capacitation and AR models making them more predictive.

## Conclusions

This work is a first step to the modelisation of whole biochemical asset of a cell, the spermatozoa, that plays a key role in the species survival. As first it was proposed to complete the dialectic of in vivo - in vitro model adding in silico model to increase the resources available to study a complex phenomenon as the physiology of male gamete. In addition the used modelling strategy could explain many biological aspects of cell function that are out of focus looking at the single molecular determinant, thus overcoming the reductionist approach which did not consider the complexity of molecules and their interactions. This could have potential application in diagnostics, therapeutics and drug discovery in the medicine of reproduction offering to the scientific community new operative resources.

## Methods

### Database realization, network construction and data analysis

At present a database containing the information about the molecular events occurring during the capacitation and AR of human or mammal spermatozoa does not exist, as a consequence a new database was realized using Microsoft Office Excel 2003. The available information was obtained from peer-reviewed papers from PubMed http://www.ncbi.nlm.nih.gov/pubmed/. As a reference were used the data concerning human spermatozoa of the latest 10 years. When the data are lacking or to fill incomplete pathways the data from other mammals, such as mouse, horse, pig, bull, etc. were used, only if confirmed by a large consensus. The freely available and diffusible molecules such as H_2_O, CO_2_, P_i_, H^+^,O_2 _were omitted, when not necessary. In some cases the record did not represent a single molecule but complex events, such as "membrane fusion" or "protein tyrosine phosphorylation" because all the single molecular determinants of the phenomenon are still unknown. More in detail the fields of the database were:

Source molecule: represents the molecule source of interaction.

Interaction: represents the nature of interaction (activation, inhibition, ...).

Target molecule: represents the molecule target of the interaction.

Biological function: represents the functional meaning or the contest of interaction (glycolysis, lipid remodelling, oxidative phosphorylation, ...).

Species: represents the species in which the interaction was described.

Reference: represents the bibliographic source of information.

Notes: represents all the notation such as the presence of synonyms or the intracellular location, if relevant, or the explanation of complex cellular events.

The data, extracted from the database, were used to build the capacitation and AR networks using the Cytoscape 2.6.3 software http://www.cytoscape.org. The networks were spatially represented using the Cytoscape Spring-embedded Layout. This program **" **is based on a force-directed paradigm. Network nodes are treated like physical objects that repel each other, such as electrons. The connections between nodes are treated like metal springs attached to the pair of nodes. These springs repel or attract their end points according to a force function. The layout algorithm sets the positions of the nodes in a way that minimizes the sum of forces in the network" [32]. The node size was proportional to the connection number and the node color gradient was dependent from the closeness centrality. This parameter is computed as: C_c_(*n*) = 1/avg(L(*n*,*m*)), were L (n, m) is the length of the shortest path between two nodes *n *and *m*. The closeness centrality of each node ranges from 0 to 1 and it is a measure of how fast information spreads from a given node to the others nodes. The red edges represent inhibition links, the green edges the activation ones, the arrows indicates the direction of the interaction. The statistical and topological analyses of networks were carried out considering the networks as directed by the Cytoscape plugin Network Analyzer http://med.bioinf.mpi-inf.mpg.de/netanalyzer/help/2.6.1/index.html.

### Networks modification

Two different computational experiments were carried out on capacitation and AR networks. As first the network representing the elements common to both the networks was realised, using the Cytoscape plugin Network Analyzer (function "Compute intersection" of "Compare two networks" menu). This new network was called C∩A (intersection of capacitation and AR).

Then new networks were built to verify the consequences of random or targeted elimination of nodes. In the first case the nodes to be eliminated were individuated by a random procedure: a number was attributed to each node and the computer was asked to generate random numbers (Microsoft Office Excel 2003 Random Generator). The correspondent node was eliminated from the network. In the second case the most linked nodes were removed from the networks.

## Authors' contributions

NB: have made substantial contributions to work conception and design and to analysis of data; MM and BB: have been involved in revising critically the manuscript for important intellectual content. All the Authors read and approved the final manuscript.
